# Host Control of Insect Endogenous Retroviruses: Small RNA Silencing and Immune Response

**DOI:** 10.3390/v6114447

**Published:** 2014-11-18

**Authors:** Marie Fablet

**Affiliations:** 1 Laboratoire de Biométrie et Biologie Évolutive, UMR5558, CNRS, F-69622 Villeurbanne, France; E-Mail: marie.fablet@univ-lyon1.fr; Tel.: +33-4-72-43-29-16; Fax: +33-4-72-43-13-88; 2 Université Lyon 1, F-69622 Villeurbanne, France; 3 Université de Lyon, F-69000 Lyon, France

**Keywords:** LTR retrotransposon, transposable element, piRNA, siRNA, piRNA cluster, *Drosophila*, mosquito, evolution

## Abstract

Endogenous retroviruses are relics of ancient infections from retroviruses that managed to integrate into the genome of germline cells and remained vertically transmitted from parent to progeny. Subsequent to the endogenization process, these sequences can move and multiply in the host genome, which can have deleterious consequences and disturb genomic stability. Natural selection favored the establishment of silencing pathways that protect host genomes from the activity of endogenous retroviruses. RNA silencing mechanisms are involved, which utilize piRNAs. The response to exogenous viral infections uses siRNAs, a class of small RNAs that are generated via a distinct biogenesis pathway from piRNAs. However, interplay between both pathways has been identified, and interactions with anti-bacterial and anti-fungal immune responses are also suspected. This review focuses on *Diptera* (Arthropods) and intends to compile pieces of evidence showing that the RNA silencing pathway of endogenous retrovirus regulation is not independent from immunity and the response to infections. This review will consider the mechanisms that allow the lasting coexistence of viral sequences and host genomes from an evolutionary perspective.

## 1. Introduction

Endogenous retroviruses (ERVs) are retroviral sequences that permanently remain in the host genome and are vertically transmitted from parent to progeny. They are relics of retrovirus infections of germline cells, which did not end up in cellular lysis and were eventually transmitted to the genome of the zygote and all cells of the subsequent organism. Due to genetic drift, certain of these retroviral insertions may reach fixation, which become ERV insertions, shared by all the individuals of the species. ERVs make up 8% of the human genome [1]; however, this mostly includes inactive and degenerated copies. In *Drosophila melanogaster*, these sequences were estimated to make up approximately 2% of euchromatin [2] and are responsible for many spontaneous mutations [3]. The canonical structure of an ERV is composed of three open reading frames (ORFs): *gag* encodes proteins of the capsid, *pol* encodes the enzymatic machinery principally for reverse transcription and integration into the host genome, and *env* encodes the proteins involved in envelope formation. These coding sequences are bordered by long terminal repeats (LTRs), which display all signals necessary for expression and reverse transcription. Due to structural similarity, ERVs are included into the LTR retrotransposon class of transposable elements (TEs) [4]. Note that the International Committee on Taxonomy of Viruses (ICTV) includes vertebrate ERVs into the *Retroviridae* family while insect ERVs belong to the *Metaviridae* family.

In this review, we focus on *Diptera* ERVs, particularly in mosquitoes and *Drosophila*, which diverged approximately 250 million years ago (Mya). The mosquitoes *Anopheles gambiae*—the main vector of malaria—and *Aedes aegypti* diverged 150 Mya. The latter carries many arboviruses (arthropod-borne viruses, reviewed in [5]). Arboviruses group into various viral families but are predominantly RNA viruses that cycle between vertebrates and hematophagous arthropod vectors. These viruses are of major concern for human health and include Dengue virus, Chikungunya virus, West Nile virus, O’nyong-nyong virus, *etc. Drosophila* is a genetic model that allows for a relatively easy deciphering of molecular mechanisms, as is also beginning to be the case for the above mosquito species, whose genomes were sequenced.

Insect ERVs have been well described for a long time and include *gypsy*, *ZAM*, *Idefix*, *tirant*, *17.6*, *297* and *nomad* in *D. melanogaster*; *Tv1* in *Drosophila virilis*; *tom* in *Drosophila ananassae*; or *Osvaldo* in *Drosophila buzzatii* [6]. These sequences are referred to as IERVs for Insect Endogenous RetroViruses or Insect ERrantiViruses and form a monophyletic group [6]. They were proposed to result from an LTR retrotransposon devoid of *env* that acquired the *env* gene of a baculovirus (dsDNA virus with no RNA stage) [7]. Like in *Drosophila*, *gypsy*-like and *Osvaldo* elements are also found in the sequenced genome of *An. gambiae* [8]. In this genome, the total genomic TE proportion of 16% is relatively low [8] compared to the sequenced genome of *Ae. aegypti*, which is made up of 47% TEs, including 10.5% LTR retrotransposons [9]. The *Culex quinquefasciatus* genome, within the *Culex pipiens* species complex, displays an intermediate value of 29% TEs, but only 4% LTR retrotransposons [10].

Natural selection favored the establishment of control pathways that allow the avoidance of the deleterious consequences of ERV reactivation and thus maintain genomic stability. Epigenetic mechanisms are involved, such as RNA silencing using piRNAs (Piwi-interacting RNAs), which were initially called “rasiRNAs” (repeat-associated small interfering RNAs) [11,12,13,14,15]. piRNA silencing is a post-transcriptional mechanism that, in addition, triggers chromatin modifications that reinforce the inhibition at the transcriptional level [16,17,18]. Exogenous viruses are silenced by siRNAs, which form a different class of small RNAs [19,20,21]. The biogenesis and silencing mechanisms of the different small interfering RNAs are usually studied independently; however, as developed below, clear evidence shows that interplay exists between them and even with other pathways of antiviral immunity. It is well described that ERVs are inhibited by piRNAs [13,15,22,23,24]. However, some studies also show the involvement of siRNAs in this silencing [25,26,27,28,29], as well as the involvement of piRNAs in the antiviral response [30,31,32,33,34]. The molecular source of piRNAs from particular genomic clusters also raises questions about the evolutionary setting of a silencing pathway dedicated to ERVs. Further, we wonder whether it is possible that these sequences are a target of the more classical immune pathways that were recently shown to be involved in the antiviral response. The focus of this review is ERV regulation and the way it is intertwined with immunity pathways. Taking advantage of recent data published on arboviruses, it will consider the mechanisms allowing the lasting coexistence of viral sequences and host genomes in an evolutionary perspective.

## 2. Endogenous Retroviruses Are Silenced by piRNAs

**Figure 1:**
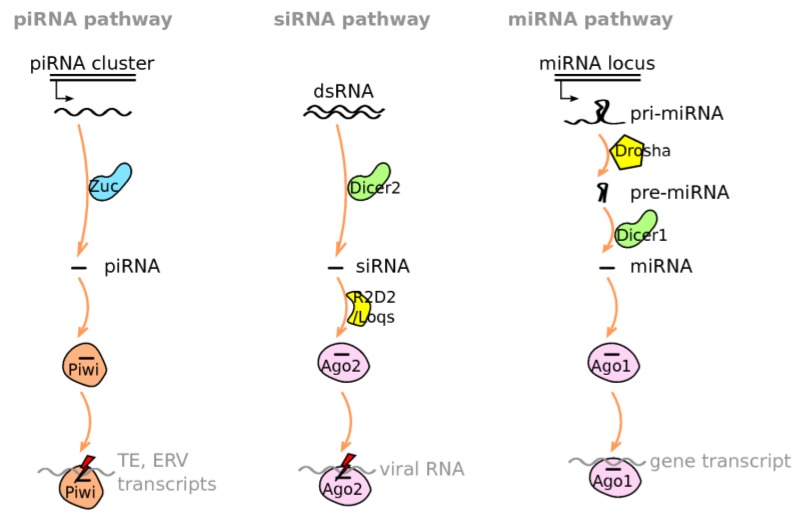
Small RNA Pathways. In somatic ovarian cells, piRNA clusters produce long transcripts that are cleaved into piRNAs by Zuc. piRNAs are then loaded onto Piwi, and the resulting complex slices TE and ERV transcripts. Please refer to Figure 2 for piRNA pathways in germline cells. siRNAs are derived from double-stranded RNAs (dsRNAs) by the action of Dicer-2. R2D2 or Loqs participate in loading siRNAs onto Ago2, and the resulting complex displays slicing activity against complementary transcripts (most often of viral origin). miRNAs derive from the transcription of pri-miRNAs that are cleaved into pre-miRNAs by Drosha, and subsequently processed by Dicer-1. miRNAs are loaded onto Ago1, and the resulting complex tunes gene expression using different mechanisms.

The molecular process of ERV silencing by piRNAs is being actively studied and is becoming well understood in *Drosophila*, and many reviews have been published on that topic [20,22,23,24,35,36]. piRNAs are produced from particular genomic loci called “piRNA clusters” [37], which will be discussed in detail in the last section of this article. piRNAs are single stranded, 23–30 nt RNAs that bind to ERV transcripts in a sequence-specific fashion and carry them to the catalytic site of the slicing Argonaute proteins [20,22,23,24,35,36]. Figure 1 illustrates the main steps of the different small RNA pathways and Table 1 recapitulates small RNA properties. 

**Table 1:** Small RNA Properties. Data in this table come from *D. melanogaster* studies. However, these properties were found to be largely conserved in mosquitoes.

Properties	piRNAs	siRNAs	miRNAs
Size	23–30 nt	~21 nt	20–22 nt
Chemical modifications	3' terminal 2'-*O*-methylation	3' terminal 2'-*O*-methylation	/
Sequence bias	1U (bound to Piwi and Aub) or 10A (bound to Ago3)	No tendency to begin with uracil	Tendency to begin with uracil
Orientation	Antisense (bound to Piwi and Aub) more abundant than sense (bound to Ago3)	Equal numbers of sense and antisense	Sense (relative to the miRNA locus)
Precursor	Single stranded, transcribed from cluster	Long dsRNA	Single stranded pri-miRNA, processed into hairpin structured pre-miRNA
Biogenesis effector	Primary pathway: Zuc	Dicer-2	Dicer-1
	Secondary pathway: Ago3, Aub		
Slicing effector	Primary pathway: Piwi	Ago2	Ago1
	Secondary pathway: Ago3, Aub		

Molecular effectors of the piRNA pathway have been particularly well described in the nematode, *Caenorhabditis elegans*, and in the fruit fly, *D. melanogaster*. Numerous proteins are required for the proper biogenesis of piRNAs; however, the Argonaute proteins are the most extensively studied [38]. This family contains the AGO and PIWI subclasses, which all display PAZ (PIWI, Argonaute, Zwille) and PIWI domains [38]. The PIWI domain allows the 'slicing' of target mRNAs due to its RNase-H activity [39]. In *D. melanogaster*, the AGO subclass is composed of Ago1 (Argonaute 1)—involved in the miRNA pathway—and Ago2 (Argonaute 2)—involved in the siRNA pathway—, and the PIWI subclass gathers Ago3 (Argonaute 3), Aub (Aubergine) and Piwi, all involved in the piRNA pathway [20,24,38].

**Figure 2:**
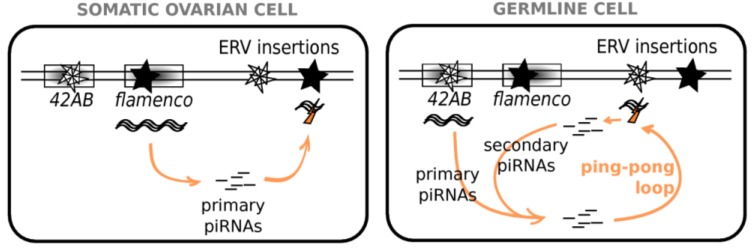
piRNA Pathways in Ovarian Cells. In somatic ovarian cells, clusters, such as *flamenco*, produce long transcripts, which are processed into primary piRNAs. These piRNAs then trigger the degradation of transcripts of the corresponding TE/ERV families, wherever copies may be integrated in the genome. In germline cells, clusters, such as *42AB* produce transcripts, which are processed into primary piRNAs. These piRNAs then trigger the degradation of transcripts of the corresponding TE/ERV families into piRNAs, which trigger the degradation of antisense transcripts also produced by clusters. This generates antisense secondary piRNAs, which are able to target TE/ERV transcripts. Such a phenomenon, called “ping-pong loop”, allows amplification of the silencing.

Two biogenesis pathways account for piRNA production (Figure 2). In the primary pathway, piRNAs called “primary piRNAs” derive from the transcription of piRNA clusters. This process involves the Piwi and Zuc (Zucchini) proteins [40], among others. In the secondary pathway, piRNAs called “secondary piRNAs” are produced and amplified according to the so-called “ping-pong” loop [37]. The initial feeding of the loop comes either from primary piRNAs or maternally deposited secondary piRNAs. Antisense piRNAs bind to Piwi or Aub, which then slice complementary sense transcripts into sense piRNAs. These latter piRNAs bind to Ago3, which slices antisense transcripts into antisense piRNAs. This leads to the so-called “ping-pong signature”, corresponding to the first 10 nucleotides of piRNAs being complementary to partner piRNAs in the opposite sense [37]. This amplification process allows a rapid and efficient response against intensely active sequences. In *D. melanogaster* ovaries, which piRNA pathway is at play depends on the cellular type [41]. In follicular somatic cells, which surround the ovary, only the primary pathway is active. In germline cells—they include 15 nurse cells and one oocyte at the end of the oogenesis process—both primary and secondary pathways are involved in piRNA production. However, the activity of each piRNA cluster is specific to either the germline or somatic ovarian cells.

*Drosophila* TEs are classified as germline or somatic [41] depending on the piRNA clusters involved. Elements targeted only by the primary pathway that, in the ovary, are expressed only in somatic cells are enriched in *gypsy* family ERVs (e.g., *gypsy*, *ZAM*) [41]. However, ERVs are also found in the other classes: elements active mainly in the germline and controlled by the secondary piRNA pathway (e.g., *17.6*) and elements with a mixed expression and a double piRNA control (e.g., *tirant*, *mdg1*) [41]. In the case of *tirant*, it must be noted that maternally deposited germline piRNAs are able to initiate the somatic repression of the ERV in the ovaries of the subsequent generation [42], which suggests that somatic and germline piRNA pathways are not independent. In addition, as exemplified by the study of *tirant*, the cellular location of the ERV transcripts within the ovary and the presence and nature of the corresponding piRNAs may vary between wild-type strains of the same species [43], which highlights the strain specificity of Malone *et al.*’s classification [41].

Mosquito genomes display all of the genes involved in piRNA pathways, and ping-pong signatures are also found in *Ae. aegypti* piRNAs, suggesting that the same amplification process is also at play in mosquitoes [44]. However, certain piRNA genes were subject to rounds of duplications in some species. The PIWI gene family was subject to an expansion in *Ae. aegypti* and *Cx. pipiens* genomes; in addition to a single *ago3* ortholog, two other distinct *ago* clades are found [45]. The *ago4* clade includes three and four genes in *Cx. pipiens* and *Ae. aegypti*, respectively, and the *ago5* clade gathers three genes in both genomes [45]. Such a gene amplification is not found in all mosquito genomes. Indeed, *An. gambiae* and *Anopheles stephensi*—another malaria vector—display one-to-one orthologs for *piwi*, *ago3* and *aub* [46]. Authors suggest that expansions in genes involved in the piRNA pathway may have been adaptive in *Ae. aegypti* and *Cx. pipiens* and allowed to address a higher amount of retrotransposons in these genomes compared to that of *An. gambiae* and *D. melanogaster* [45]. Genes involved in the piRNA pathway were shown to evolve quickly in *Drosophila* [47,48,49,50]. In addition, sequence analysis of the *ago* and *piwi* genes indicates that they are evolving faster in *Ae. aegypti* and *Cx. pipiens*, which are major arbovirus vectors, than in *An. gambiae* and *D. melanogaster* [45].

## 3. siRNAs Are Involved in Antiviral Defense

In addition to piRNAs, two other small RNA classes are well characterized in *D. melanogaster*: siRNAs and miRNAs. What principally distinguishes piRNA biogenesis from siRNA biogenesis is that piRNAs are produced from single-stranded precursors, independently of Dicer [20]. Like siRNAs, the production of miRNAs is Dicer-dependent. miRNAs are encoded in the genome and derive from the transcription of primary miRNAs (pri-miRNAs) that are cleaved into shorter precursors (pre-miRNAs) by Drosha. They are subsequently processed by Dicer-1, and the inhibition of translation and/or degradation of complementary transcripts is achieved by Ago1 [20,24] (Figure 1). miRNAs are implicated in tuning cellular gene expression [20]. Doing so, they may also participate in immune pathway regulation, and viruses were also found to encode miRNAs (see [51] for a review on miRNA roles in viral infection).

The siRNA pathway is clearly implicated in antiviral defense in insects [30,52,53,54]. This immune response is triggered by Dicer-2 recognizing intracellular, long, double-stranded RNAs (dsRNAs) produced by a variety of viruses [55] and cutting them into siRNAs. siRNAs bind Ago2, and only one strand of the duplex is maintained. The dsRNA-binding proteins Loquacious (Loqs) or R2D2 participate in loading siRNAs onto Ago2. The siRNA-Ago2 complex, also called RISC (RNA-induced silencing complex), recognizes complementary transcripts, and Ago2 cleaves them (see [19,20] for reviews of the siRNA pathway) (Figure 1). Antiviral response may spread due to double-stranded viral RNAs generated in infected cells being able to enter uninfected cells where they then trigger the siRNA pathway [56]. A fraction of siRNAs are also found to be of endogenous origin and are referred to as “endo-siRNAs” [20]. However, their biochemical structure and their mode of action are roughly similar to those described for siRNAs of viral origin. Therefore, throughout this review, the acronym “siRNA” will be used in both cases.

Like the piRNA pathway genes, genes involved in the siRNA pathway appear to be subject to rapid evolutionary dynamics. *dcr-2* (encoding Dicer-2) and *ago2* are subject to a very rapid evolution in *D. melanogaster* [57]. *ago2* is a single locus in *Ae. aegypti*, *An. gambiae* and *D. melanogaster*, but it is present in two copies in *Cx. pipiens* [45]. A population study of *Ae. aegypti* detected rapid, positive, diversifying selection in effectors of the siRNA pathway [58]. On the contrary, genes involved in the miRNA pathway, such as *dcr-1* (encoding Dicer-1) and *ago1*, also appear to undergo rapid evolution in *Ae. aegypti*—although slightly less pronounced than in the case of the siRNA pathway—[58], but do not evolve more rapidly than the genome average in *D. melanogaster* [57].

## 4. Interplay between the Different Small RNA Classes

The different classes of small RNAs are regularly presented as independent. However, more and more studies suggest that interactions exist. In particular, it appears that piRNAs are not only involved in ERV or, more broadly, in TE silencing. Instead, they may have various targets. For instance, piRNAs can participate in the regulation of cellular gene expression. 3' untranslated regions (UTRs) of cellular genes can produce piRNAs in the follicular cells of *Drosophila* [59,60]. piRNAs derived from TEs have also been found to participate in cellular gene regulation during development [61]. In addition, piRNAs are not restricted to ERVs; they can also be involved in classical antiviral defense, as was shown in *Drosophila* [31] and in *Aedes* in the cases of the Semliki Forest virus [32], Dengue virus [33] or Sindbis virus [34]. For *An. gambiae*, Keene *et al.* [30] showed that inhibition of *ago2*—involved in the siRNA pathway—as well as *ago3*—involved in the piRNA pathway—increased O'nyong-nyong virus titers. 

Conversely, ERVs are not exclusively controlled by piRNAs; they are also the target of some siRNAs. Immunoprecipitation of Ago2-bound small RNAs in *D. melanogaster* ovaries revealed 53% of sequences matched TEs [25]. Depletions in *dcr-2* and *ago2* cause an increase in retrotransposon transcripts in somatic cells [26,27]. Since *dcr-2* and *ago2* participate in the siRNA but not the piRNA pathway, this indicates that siRNAs are also involved in the silencing of ERVs. As such, some authors have suggested that the positive selection acting on the effectors of the siRNA pathway in *Ae. aegypti* may be a response to TE activity [58]. In ovarian somatic cells, where only the primary piRNA pathway is active, Lau *et al.* [28] found that TEs are targeted by both piRNAs and siRNAs but in relative proportions that are TE-family specific. For instance, *mdg1* and *Idefix* produce almost no siRNAs, whereas *gypsy* preferentially generates siRNAs over piRNAs. Using somatic cells (S2 cells and *Drosophila* heads), Ghildiyal *et al.* [29] found siRNAs corresponding to the three main types of TEs, *i.e.*, LTR retrotransposons, non-LTR retrotransposons and transposons. However, LTR retrotransposons were overrepresented, even after accounting for their higher abundance in the genome. It is to be noted that LTR retrotransposons are precisely the TE class of ERVs. Kawamura *et al.* [26] also found that ERV *297* was overrepresented among siRNAs in *D. melanogaster* S2 cells (of somatic type). Even more complex scenarios may be imagined because some authors also found pi-siRNA ping-pong pairs corresponding to retrotransposon sequences [62,63]. Moreover, it has been demonstrated that piRNAs and siRNAs can arise from the same genomic clusters, which suggests that they can arise from the same single precursor transcript [25,29,62]. A direct functional interaction between piRNA and siRNA pathways has also recently been demonstrated to exist in the nematode *C. elegans* [64]. Bagijn *et al.* [64] showed that piRNAs silence endogenous transcripts by triggering a secondary siRNA response. This secondary siRNA amplification could ensure the effective silencing of abundant targets, as the ping-pong loop does in insects. In *D. melanogaster*, compensation for mutated primary or secondary piRNA pathways via an increase in siRNA production was reported in the case of numerous elements including the *ZAM* ERV [16].

As an explanation for the relative involvements of these different pathways, some authors suggest that ERVs are targeted by piRNAs in the gonads and siRNAs in the soma [29]. Indeed, in the very early embryo, which reflects the maternal germline RNA content, piRNAs are the major class of TE-directed small RNAs, and they are progressively replaced by siRNAs during development [65]. However, siRNAs can be found in germline cells [66], and piRNAs of viral origin with ping-pong signatures are detected in *Aedes* somatic cells [67]. Although less abundantly than in ovaries or embryos, small RNAs displaying the features of germline piRNAs are also found in *D. melanogaster* heads and imaginal discs [68,69]. Nevertheless, it must be noted that PIWI proteins are only expressed in the gonads in *Drosophila*, while they are additionally found in the soma of *Aedes* mosquitoes, at least in the case of viral infection [67].

**Figure 3:**
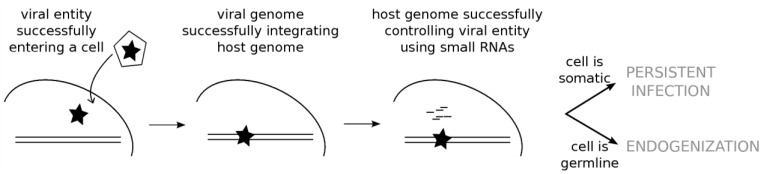
Endogenization and Persistent Infections Originate from Comparable Mechanisms. Endogenization of retroviral sequences results from the infection of a germline cell by a minimally aggressive retrovirus that subsequently remained in the genome and reached fixation in the species. The persistent infection state, as frequently observed in the case of arboviruses, is the consequence of the stable genomic integration of the virus, which remains silent in the insect cell. Both phenomena differ mainly in the nature of the infected cells, either somatic or germline.

ERVs and exogenous viruses both can be the targets of piRNAs as well as siRNAs, and this raises the question of whether and how the cell distinguishes between endogenous and exogenous viral entities. In *Drosophila* S2 cells, Goic *et al.* [70] worked on persistent infections of the Flock House virus. Contrary to acute infections, persistent infections correspond to the absence of cellular lysis and the viral genome remaining within the cell without being cleared by the immune system of the host. Goic *et al.* showed that persistent infections of the Flock House virus are due to viral integrations into LTR retrotransposon sequences thanks to the retrotransposon machinery. Chimerical siRNAs can subsequently be produced. In this way, persistent infections are allowed by viral integration into the host genome. This draws a parallel between ERVs and arboviruses, which frequently display persistent infections in Arthropods. Comparable processes may be at play in both persistent infection and endogenization, the difference coming from the nature of the infected cell, somatic or germline, respectively (Figure 3). This results in the ERVs establishing a long lasting cohabitation with insect genomes, whereas persistent infections of arboviruses most often imply new infections at each generation.

## 5. Endogenous Retroviruses and Innate Immunity

Antiviral immunity in Arthropods has long remained an unsolved question. For less than a decade, we have known that immune pathways classically dedicated to bacterial and fungal infections are also at play against viruses. These are the Toll, Immune deficiency (Imd) and Jak-STAT pathways, which are nicely reviewed by Kingsolver *et al.* [21]. The Toll pathway is classically considered to be the immune pathway acting against Gram-positive bacteria and fungi [21]. Through a serine protease cascade, the NF-κB-like transcription factors, Dorsal and Dif, are translocated to the nucleus and promote the expression of multiple antimicrobial peptides (AMPs) including Drosomycin. The Imd pathway is thought to be dedicated to Gram-negative bacteria [21]. In this case, the NF-κB-like transcription factor Relish is activated, leading to the expression of several AMPs including Diptericin. These pathways are conserved in mosquitoes where the orthologs of *dorsal* and *relish* are *rel1* (which is further duplicated in *An. gambiae*) and *rel2*, respectively [71]. However, these authors also found that *dif* was absent from both *Ae. aegypti* and *An. gambiae*. The Jak-STAT pathway is also involved in the antibacterial and antiviral responses by regulating the production of AMPs [21]. The Domeless receptor interacts with a Janus kinase (Hopscotch), which activates STAT. The activated STAT then moves to the nucleus and promotes the transcription of downstream effector genes [21].

The implications of these pathways in the antiviral response are not systematic, since none of these were found to be activated in sigma virus infections in *D. melanogaster* [72]. Nevertheless, depending on the viral agent, distinct pathways may be triggered (see [73,74] for reviews). The Toll pathway is involved in response to *Drosophila* X virus [75] and Dengue virus in *Ae. aegypti* in addition to the Jak-STAT pathway [76]. The Jak-STAT but not the Toll nor the Imd pathways are activated during *Drosophila* C virus infections [77]. Response against Sindbis virus infection is mediated by the Imd, and potentially the Jak-STAT pathways, but not the Toll pathway in flies and mosquitoes [78]. However, although the Toll, Imd or Jak-STAT pathways are classically characterized as being involved in the antibacterial and/or antifungal response, viruses do not present bacterial nor fungal PAMPs (pathogen associated microbial patterns), which raises the question of the specific mechanism that allows activation of these pathways. Some authors have proposed that the activation of these pathways occurs within the cell and could be mediated by a cytoplasmic sensor of viral RNA, such as Dicer-2 [78]. Indeed, although the siRNA pathway is not directly involved, it was shown that Dicer-2 is responsible for the activation of *vago*, a gene whose product was demonstrated to control viral load [79]. Vago was also demonstrated to act against West Nile virus infections in *Culex* by activating the Jak-STAT pathway [80].

It is to be noted that ERVs derive from RNA virus infections and that, so far, all known exogenous viruses naturally infecting *D. melanogaster* are RNA viruses [73,81]. Currently, no study reports activation of the Toll, Imd or Jak-STAT pathways directed against ERVs, even if viral particles may be produced and involved in the oocyte infection [82,83,84]. Without anticipating results, we suggest that the primary explanation for this observation is that it has never been looked for. If the PAMP implicated in the antiviral response is intracellular viral RNA, there is no theoretical impediment that such an immune response may also concern ERVs.

## 6. piRNA Genomic Clusters

Among these various antiviral response pathways, our best-established knowledge regarding ERVs comes from the piRNA pathway in *D. melanogaster*. piRNAs that silence TEs and ERVs are produced by particular regions of the genome that are specific for either the somatic or germline inhibition pathways [37,41]. Major clusters in *Drosophila* are *flamenco* (~180 kb locus at the boundary between euchromatin and pericentromeric heterochromatin on the X chromosome), which produces somatic piRNAs from a single long antisense transcript, and the locus named “*42AB*” after its cytological position, which produces germline piRNAs of both orientations [37,41]. *flamenco* is principally involved in the silencing of the *gypsy*, *ZAM* and *Idefix* ERVs, whereas *42AB* targets a broader diversity of TEs [37]. *flamenco* therefore appears as a piRNA cluster particularly dedicated to ERVs, but it may also contain other classes of elements [85]. An analysis of *Ae. aegypti* piRNA clusters showed that they make up ~20% of the assembled genome, which is higher than the observed 3.5% for *D. melanogaster* clusters; however, they are not enriched in TE sequences compared to the rest of the genome [44]. Importantly, it must be noted that eight of the 30 top piRNA clusters detected in *Ae. aegypti* are likely to be of viral origin [44]. Because this species harbors many arboviruses, we speculate that this cluster sequence composition could be the result of successive rounds of viral integrations allowing the persistent infection state observed in the case of arboviruses, as described by [70]. The fact that these sequences are maintained in the mosquito genome implies that they once integrated into the germline cell genome—and subsequently reached fixation.

It has been demonstrated that the presence of the major clusters is relatively well conserved across *Drosophila* species, indicating their ancient origin [41]. In *Drosophila yakuba* and *Drosophila erecta*, *flamenco* loci were identified at the same positions as in *D. melanogaster*. Identically, they are enriched in LTR retrotransposons of the *gypsy* family, although the precise sequences differ between species [41]. In addition, the uniform orientation of TE insertions is maintained in *flamenco* in these different species [41], which ensures production of antisense primary piRNAs. Zanni *et al.* [85] found numerous rearrangements, deletions and segmental duplications ranging in size from several base pairs up to several kb, as well as losses and gains of TEs in the *flamenco* locus using a panel of three *D. melanogaster* strains. This reveals the highly dynamic nature of such a locus, even at the intra-species scale. Conservation across millions of years and high intra-species variability seem incompatible unless we consider that it is the structure of the loci that is conserved along evolution, ensuring their properties of trapping TEs, whose sequences are expected to be different between species. These TE trap properties may be ensured by particular chromatin modifiers (as the Rhino-Deadlock-Cutoff complex does in germline piRNA clusters [86], see below).

Zanni *et al.* [85] demonstrated that the “permissive” state of a strain regarding a given ERV—*i.e.*, the fact that the ERV is relatively active and transposes in this strain—is due to the absence of its sequence in the *flamenco* locus. New TE insertions in piRNA clusters allow for the production of the corresponding piRNAs and provide the control machinery against the whole TE family wherever individual copies may be inserted [87]. Therefore, some authors evoke the parallel between the piRNA silencing pathway and adaptive immune systems [37,41,85]. RNA silencing can thus be considered to be “genomic immunity”. Vermaak *et al.* [88] proposed a model in which the Rhino protein of the HP1 family—heterochromatic proteins—might interact with the integration machinery of TEs to direct their insertion into heterochromatin and, more specifically, into piRNA clusters. Rhino was further demonstrated to be associated with dual-strand clusters, such as *42AB,* and not with single-strand clusters, such as *flamenco* [89]. In a complex with Deadlock and Cutoff, Rhino allows the particular transcription of germline dual-strand clusters (5' end protection of nascent transcripts and suppression of termination) [86].

**Figure 4:**
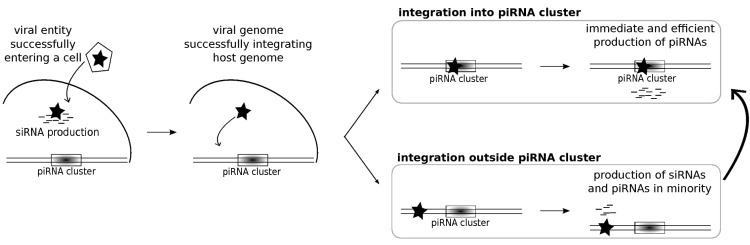
Schematic View of Small RNA Control Setup. Consider an RNA-based viral entity that managed to escape antiviral response, such as Toll, Imd or Jak-STAT pathways. When its genetic material is released into the cell, siRNAs are produced and trigger its degradation. Despite this siRNA defense, we can imagine that some viral genetic material may become integrated into the cellular genome (either by its own machinery or with the help of endogenous enzymes encoded by ERVs or other TEs). Integration into an already existing piRNA cluster, such as *flamenco*, provides immediate cellular protection through piRNA production. Immune memory against the corresponding viral family is acquired. In case the integration happens elsewhere in the genome, siRNAs are produced from that loci, as well as piRNAs, although in the minority. Control by siRNAs is not absolute, which allows subsequent insertions into a major piRNA cluster, which then provides complete control.

In addition to these major clusters, many minor clusters are found all along the genome [37]. It has recently been demonstrated that a new, active insertion of a TE in euchromatin becomes a source locus for germline piRNAs as well as siRNAs and that small RNA production spreads into the flanking regions of the insertion [63]. This was observed for active insertions, meaning those that are young and thus potentially polymorphic between populations. Mohn *et al.* [86] showed that such TE insertions displayed the same particular transcription behavior as that observed for germline piRNA clusters (5' end protection of nascent transcripts and suppression of termination). This raises the question of the possibility that a new retroviral insertion also becomes a piRNA source locus. Analysis of recent retroviral endogenization events would provide clues to this issue. Unfortunately, they have seldom been reported [90]. In a very interesting study, Rozhkov *et al.* [66] introduced an active *Penelope* TE into the naive *D. melanogaster* genome. (Note that *Penelope* comes from *D. virilis* and belongs to a particular class of retrotransposons, distinct from LTR and non-LTR retrotransposons.) They observed that although piRNAs were produced against the already setup TE families, *Penelope* was initially mostly targeted by siRNAs rather than piRNAs. Therefore, it appears that in the first steps of viral infection or TE mobilization, the siRNA pathway is the first line of defense, allowing survival of the organism. Subsequently, the piRNA pathway comes into play against those integrated sequences that might disturb genomic stability and threaten subsequent generations. Rozhkov *et al.* [66] also observed that the siRNA control of *Penelope* was incomplete such that the TE was still able to transpose. This may allow new transposition events to occur in a major piRNA cluster, thus establishing an efficient and long lasting silencing of the family [91] (Figure 4). This is increasingly efficient as the piRNA pathway also triggers transcriptional silencing via sequence-specific heterochromatinization [16,17,18]. We speculate that the relative intensities of the piRNA control increases over the siRNA control as time passes from the integration time. While siRNA control is immediately engaged in case of a viral attack, regulation using piRNAs is expected to be more frequent for sequences displaying a long-term established cohabitation with the host genome. 

## 7. Conclusions

Some authors have shown that the host microbiota plays an important role in the defense against pathogens because it allows the induction of a basal level of immune activity [76]. In the same way, it is tempting to propose that ERVs may also be involved in antiviral defense by maintaining RNA silencing pathways. Otherwise, one could think that trade-offs may exist between the ability to control ERV sequences and the ability to counteract viral attacks. Observed natural variability in responses to viral infection may reflect various ERV contents. Alternatively it may reflect the variability in the sequences of piRNA cluster loci, which is most often not studied in classical differential expression experiments. Indeed, this genomic immune memory provided by piRNA clusters may be of fundamental importance when dealing with viral infections. It is a good bet that studies taking advantage of the observed natural variability in ERV contents and regulation will provide answers [42,43,92].
